# Best practice for passaging murine embryonic enteric neuronal cell line before differentiation

**DOI:** 10.1007/s10616-016-9953-6

**Published:** 2016-02-24

**Authors:** Carmen D. Rietdijk, Lydia de Haan, Richard J. A. van Wezel, Johan Garssen, Aletta D. Kraneveld

**Affiliations:** 1 Division of Pharmacology, Faculty of Science, Utrecht Institute for Pharmaceutical Sciences, Utrecht University, Universiteitsweg 99, 3584 CG Utrecht, Netherlands; 2 Division of Pharmacoepidemiology and Clinical Pharmacology, Faculty of Science, Utrecht Institute for Pharmaceutical Sciences, Utrecht University, Universiteitsweg 99, 3584 CG Utrecht, Netherlands; 3 Department of Biomedical Signals and Systems, MIRA, University of Twente, Drienerlolaan 5, 7522 NB Enschede, Netherlands; 4 Department of Biophysics, Donders Institute of Brain, Cognition, and Behaviour, Radboud University Nijmegen, 6525 EZ Nijmegen, Netherlands; 5 Nutricia Research, Utrecht Science Park, Uppsalalaan 12, 3584 CT Utrecht, Netherlands

**Keywords:** Murine immorto fetal enteric neuronal cell line, IM-FEN, Neurons, Growth rate, Seeding density, Enteric nervous system, Best practice

## Abstract

The enteric nervous system (ENS) is a complex network of neurons in the gut, regulating many local, vital functions of the gastro-intestinal tract. The ENS is also part of the bidirectional gut-brain axis. The murine immorto fetal enteric neuronal (IM-FEN) cell line was chosen as a model to study enteric neurons. This cell line can be differentiated into cells with a neuronal phenotype, although they do not produce action potentials in vitro. It was concluded that the differentiation process in our laboratory was successful, based on positive staining for neuronal proteins. Proliferating IM-FEN cells have an unstable growth rate in our laboratory. An indicator of growth rate was calculated, and this indicator was found to be related to seeding density and number of days in culture, and was unrelated to person culturing, previous overconfluency or passage number. The indicator of growth rate was also unrelated to successful use of differentiated cells in follow-up experiments. We recommend the following conditions for optimal culture of IM-FEN cells. Keep cells in culture until 80 % confluent before passaging, seed cells at a density of 0.0133 million cells per cm^2^, and anticipate on unstable growth rates and the risk for overconfluency.

## Introduction

The ENS is a complex autonomic network of neurons and glial cells in the gut, discovered in the nineteenth century (Furness 2005; Johnson et al. 2012). The ENS is organized in two ganglionated plexus the myenteric or Auerbach’s plexus, and the submucuous or Meissner’s plexus. The myenteric neurons lie between the inner circular and outer longitudinal smooth muscle layers, and the submucuous neurons lie in the submucosa. Together these plexus regulate gastric acid secretion, fluid motion across the epithelium, epithelial barrier integrity, local and systemic inflammatory responses, gut motility, blood flow, and the secretion of neurotransmitters, hormones and peptides in the gastro-intestinal tract (Furness 2005; Johnson et al. 2012; de Jonge 2013). The ENS communicates in a bidirectional fashion with the central nervous system through vagal and spinal autonomic nervous system pathways, the hypothalamic–pituitary–adrenal axis, and in the central nervous system the nucleus of the solitary tract and the dorsal motor nucleus of the vagus (de Jonge 2013). This connection is known as the gut-brain axis and it integrates neural, hormonal and immunological signaling.

The ENS can be studied in vitro through enteric neuronal cell culture. Primary enteric neuronal cell culture requires the sacrifice of laboratory animals, is time consuming and yields only few cells. Therefore, a cell line of enteric neurons was selected to conduct experiments, known as the IM-FEN cell line as described by Anitha et al. (2008). The IM-FEN cell line was developed at Emory University using H-2K^b^-tsA58 transgenic mice (Jat et al. 1991). These mice and the derived cells have stably integrated the thermolabile strain of simian virus 40 large tumor antigen gene. This is an immortalizing gene under the control of an interferon-γ (IFN-γ)-inducible H-2K^b^ promotor. This gene produces conditionally immortalized cells. When IM-FEN cells are cultured at 33 °C and in the presence of IFN-γ, the activity of the promotor is increased above basal levels and the gene product is active, resulting in cell proliferation (Anitha et al. 2008). Once the desired amount of cells has been reached and differentiation of the cells to a neuronal phenotype is desired, the IM-FEN cells are cultured at 39 °C without IFN-γ (Anitha et al. 2008). In the absence of IFN-γ the promotor is not stimulated and at 39 °C the gene product is inactive, inhibiting cell proliferation and supporting differentiation into neuronal cells.

The neuronal phenotype of differentiated IM-FEN cells has been thoroughly studied (Anitha et al. 2008, 2010). Differentiated IM-FEN cells express several neuronal markers (Anitha et al. 2008). Specifically they express intermediate filaments Nestin and peripherin, the neurotrophic factor receptor cRET, the serotonin receptors 2a, 3a, and 4a, the serotonin transporter SERT, the microtubule-associated protein Tau, the synaptic marker synaptophysin, and ubiquitin carboxy-terminal hydrolase L1 known as protein gene product 9.5 (PGP9.5). Differentiated IM-FEN cells express only low amounts of markers for glial cells (GFAP, S-100β) and smooth muscle cells (αSMA). Bone morphogenetic protein 2 and low dose lipopolysaccharide are able to influence the phenotype or viability of the cells (Anitha et al. 2010, 2012).

There is limited knowledge on the electrophysiological properties of the IM-FEN cell line. The firing of action potentials is the main characteristic of neurons, and to fire action potentials neurons need ion channels. Differentiated IM-FEN cells express mRNA for sodium, potassium and chloride ion channels, but unfortunately the IM-FEN cells do not seem to be able to fire action potentials in vitro (Hawkins et al. 2013). In this context it is interesting to consider that fact the IM-FEN cells were able to survive and function properly in three different in vivo experiments (Anitha et al. 2008; Raghavan et al. 2011). Mice with a reduced number of enteric neurons in the colon (Piebald heterozygous mice) were shown to have improved colonic neuronal function after IM-FEN transplantation (Anitha et al. 2008). Mice known to have impaired relaxation in the lower esophageal sphincter, pyloric sphincter and the ileum (nNOS knock out mice) were shown to have improved relaxation of the longitudinal muscle of the proximal colon after IM-FEN transplantation (Anitha et al. 2008). And when IM-FEN cells were combined with smooth muscle cells to construct an artificial internal anal sphincter, the internal anal sphincters were shown to have proper neuronal functioning when implanted in immune suppressed recombination-activation gene 1 knock out mice (Raghavan et al. 2011). Additionally, the constructed internal anal sphincters expressed markers for both excitatory and inhibitory motor neurons, and expressed neuronal β-III tubulin. These in vivo results have demonstrated that the IM-FEN cells can develop into functional neurons, and they seem to hold the promise that there are circumstances that allow the IM-FEN cells to become mature, electrically active neurons. Unfortunately at this moment it is not yet possible to culture electrically active IM-FEN cells in vitro.

Successful differentiation of IM-FEN cells was achieved in our laboratory, which was confirmed by positive staining for neuronal proteins. In our hands IM-FEN cells have an unstable growth rate during the proliferation phase. In the past this problem has also occurred in the laboratory in Atlanta where these cells were developed (personal communication). Because the cells have been growing at an unstable rate it has been hard to plan experiments or to build up a bank of frozen cells. It has been impossible to use the growth rate to judge whether the cells are healthy or not, and the unstable growth rate has increased the risk of overconfluency since unexpected spurts in growth rate have happened. In an effort to explain and find a solution to the variability in growth rate and predict growth rate in future cultures, a calculated indicator for growth rate was combined with information on the passage number, previous overconfluency (since the moment of thawing), number of days in culture (seeding to harvesting), the person culturing, and seeding density in a preliminary study. The relationship between the indicator for growth rate and the use of differentiated cells in experiments has also been examined, to determine whether the growth rate is related to suitability of the cells for experiments.

## Materials and methods

### Reagents cell culture

Dulbecco’s modified Eagle’s medium: nutrient mixture F12 (DMEM/F12) medium (Invitrogen, Bleiswijk, the Netherlands; 11330-032), glial cell line-derived neurotrophic factor (GDNF, R&D Systems Europe, Abingdon, UK; 512-GF), fetal calf serum (FCS, Bodinco BV, Alkmaar, the Netherlands), IFN-γ (Millipore, Amsterdam, the Netherlands; IF005), Penicillin/Streptomycin (Sigma Aldrich, Zwijndrecht, the Netherlands; P0781), Neurobasal A medium (Invitrogen; 10888-022), B27 supplement (Invitrogen; 17504-044), glutamine (Invitrogen; 25030-032), Trypsin/EDTA (Gibco, Bleiswijk, the Netherlands; 25300-062), selenium (Sigma Aldrich; S5261), putrescine (Sigma Aldrich; P7505), progesterone (Sigma Aldrich; P6149), insulin (Sigma Aldrich; I6634), transferrin (Sigma Aldrich; T5391), fetuin (Sigma Aldrich; F3385), bovine serum albumin (Sigma Aldrich; A8806). Antibodies: anti-PGP9.5 (Millipore; AB5925), anti-Tubulin (Covance, Rotterdam, the Netherlands; MRB-435P), anti-HuD (Millipore; AB5971), anti-peripherin (Millipore; AB1530). Secondary antibody: Alexa fluor 594 conjugated (LifeTechnologies, Bleiswijk, the Netherlands; A-21207).

### In vitro culture of IM-FEN cells

The IM-FEN cell line was established and characterized as described previously (Anitha et al. 2008). For proliferation cells were plated onto plastic 75-cm^2^ or 175-cm^2^ flasks in modified N2 medium (Heuckeroth et al. 1998) containing GDNF (100 ng/ml), FCS (10 %), penicillin/streptomycin (1 % of stock; 100 units penicillin and 100 μg streptomycin/ml) and recombinant mouse IFN-γ (20 units/ml). The cells were cultured in a humidified incubator containing 10 % CO_2_ at the permissive temperature 33 °C. The cells were observed regularly for signs of proliferation and were passaged when the flask became 80 % or more confluent, using trypsin/EDTA.

When cells were approximately 60 % confluent and proliferation was desired the medium of the cells was changed. Differentiating cells were cultured in Neurobasal-A medium containing GDNF (100 ng/ml), FCS (1 %), penicillin/streptomycin (1 % of stock; 100 units penicillin and 100 μg streptomycin/ml), B27 (1:50 dilution), and glutamine (1 mmol/l), and were placed in a humidified incubator containing 5 % CO_2_ at the non-permissive temperature 39 °C.

The calculation of the indicator of growth rate was performed on data of proliferating cells, because during proliferation the cells are growing, regularly passaged and counted. Once cells are differentiating they barely grow, and they cannot be passaged or counted anymore. Therefore analysis of growth rate during differentiation was not performed. For neuronal protein staining the cells were differentiated for 7 days.

### Immunocytochemistry neuronal proteins

Differentiated IM-FEN cells were stained for four different neuronal proteins: postmitotic neuronal marker HuD, peripheral nervous system cytoskeletal protein peripherin, neuronal microtubule protein β III tubulin, and the ubiquitin-protein hydrolase PGP9.5. The cells were washed, fixed, permeabilized and blocked before overnight incubation with primary antibody at 4 °C. Concentrations of the antibodies were for anti-PGP9.5 1:500, for anti-Tubulin 1:100, for anti-HuD 1:100 and for anti-peripherin 1:100. The secondary antibody was conjugated to Alexa Fluor 594. After immunofluorescent staining the samples were counterstained with Hoechst (Thermo Fisher Scientific, Landsmeer, the Netherlands; 62249) and preserved in ProLong Gold (LifeTechnologies; P36930). These experiments were performed once, using the first differentiated IM-FEN cells, to determine the success of our culturing techniques.

### Data analysis

The indicator for growth rate of the cells was calculated based on cell count numbers obtained when the proliferating cells were passaged, and the number of cells that were initially seeded in the flask(s), and expressed as increase or decrease of cells per day of culture.

The indicator for cell growth rate was calculated using Eq. 1, where r = indicator for growth rate, #1 = number of cells at seeding, #2 = number of cells at harvesting, t = number of days between seeding and harvest (time), ln = natural logarithm.

1 $$ {\text{r}} = { \ln }\left( {\# 2/\# 1} \right)/{\text{t}}\, \left(1/\text{day}\right)$$

These data were combined with information on the passage number of the cells at seeding, overconfluency of the cells in previous cultures (continuous cultures, not separated by a freeze–thaw cycle), the number of days between seeding and harvesting, the person culturing the cells, and the seeding density using Excel and prepared for analysis in SPSS version 21.

The range of the passage number was 33–56 (mean 43.5778), the range of number of days between seeding and harvesting was 1–5 (mean 2.7556), and the range of seeding density was 0.4286 × 10^−2^ to 2.2000 × 10^−2^ (mean 1.140644 × 10^−2^) million cells per square centimeter. The range of growth indicator was −0.649641 to 0.910212 (mean 0.22676354).

Cells in the first cycle after thawing and infected cells were excluded from the analysis. Each case consists of one flask of cells from the moment the cells are inserted in the flasks, until the moment the cells are removed from the flask. Incomplete datasets were deleted listwise, leaving 42 complete datasets suitable for analysis.

An exploratory linear regression model was built to examine the relationship between passage number, previous overconfluency, number of days in culture, person culturing, and seeding density with the indicator for growth rate of the IM-FEN cells during proliferation. Dummy variables were made for ‘person culturing’ and ‘previous overconfluency’. For ‘person culturing’ the values 0 and 1 were used to represent two individual researchers who cultured the cells independently (example: ‘0’ represents Marc, ‘1’ represents Emily), neither represent the same cells being cultured by more than one person at the same time. For ‘previous overconfluency’ the value 0 represented cells that had not been overconfluent in a continuous culture from the moment the cells were thawed, while the value 1 represented cells that had been overconfluent.

Effect sizes were expressed as standardized β coefficients, including significance and 95 % confidence intervals. Partial regression plots were made to visualize the correlation between each individual predictors and the indicator of growth rate.

For the 42 included cases, data were also collected on the final fate of the cells. Possible outcomes were proliferation, differentiation, and freezing. We were interested in the differentiated IM-FEN cells, which were 9 cases in total (21.4 % of all 42 cases). Differentiated IM-FEN cells were either used in an experiment (5 cases; 11.9 % of all cases) or discarded because of premature death (4 cases; 9.5 % of all cases), as determined by entries in laboratory journals. The relationship between the indicator of growth rate and the final fate of differentiated cells was analyzed using a Mann–Whitney *U* test.

All statistical tests were two-sided. Effects with *p* < 0.05 and confidence intervals that did not contain zero were considered significant.

## Results

**Fig. 1:**
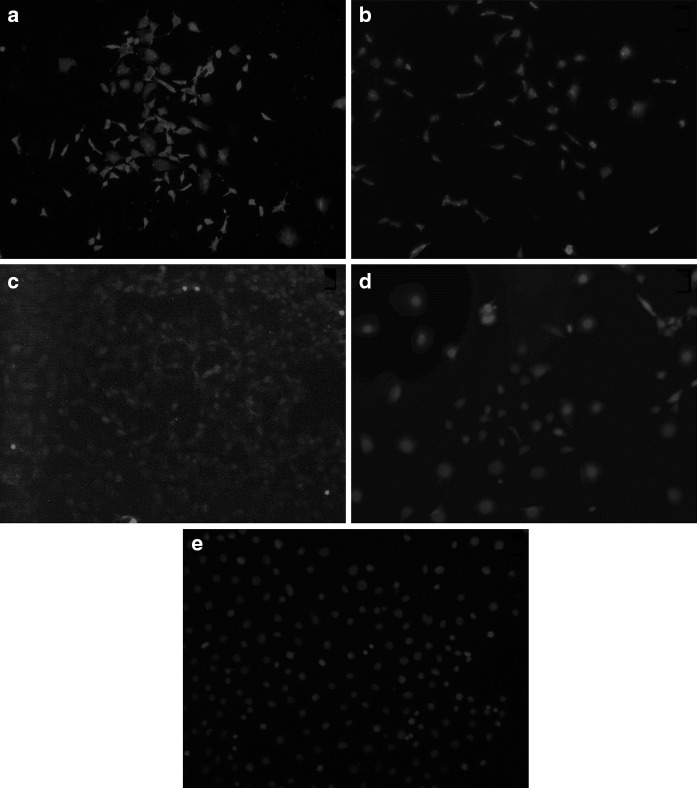
Immmunocytochemical staining for neuronal proteins (*red*) of differentiated IM-FEN cells. With Hoechst counterstaining for nuclei (*blue*). **a** HuD, **b** peripherin, **c** β III tubulin, **d** PGP9.5, **e** negative control (no antibody against neuronal protein). Cells express all four neuronal proteins, while the negative control does not show staining. (Color figure online)

Differentiated IM-FEN cells were immunoreactive to four neuronal proteins (Fig. 1a–d). The strongest reactivity was found for postmitotic neuronal marker HuD and peripheral nervous system cytoskeletal protein peripherin (Fig. 1a, b). Staining for neuronal microtubule protein beta III tubulin was weak and diffuse, but detectable (Fig. 1c). Staining for the ubiquitin-protein hydrolase PGP9.5 was detectable, but the image was blurry (Fig. 1d). No staining was detected without primary antibody (Fig. 1e).

**Fig. 2:**
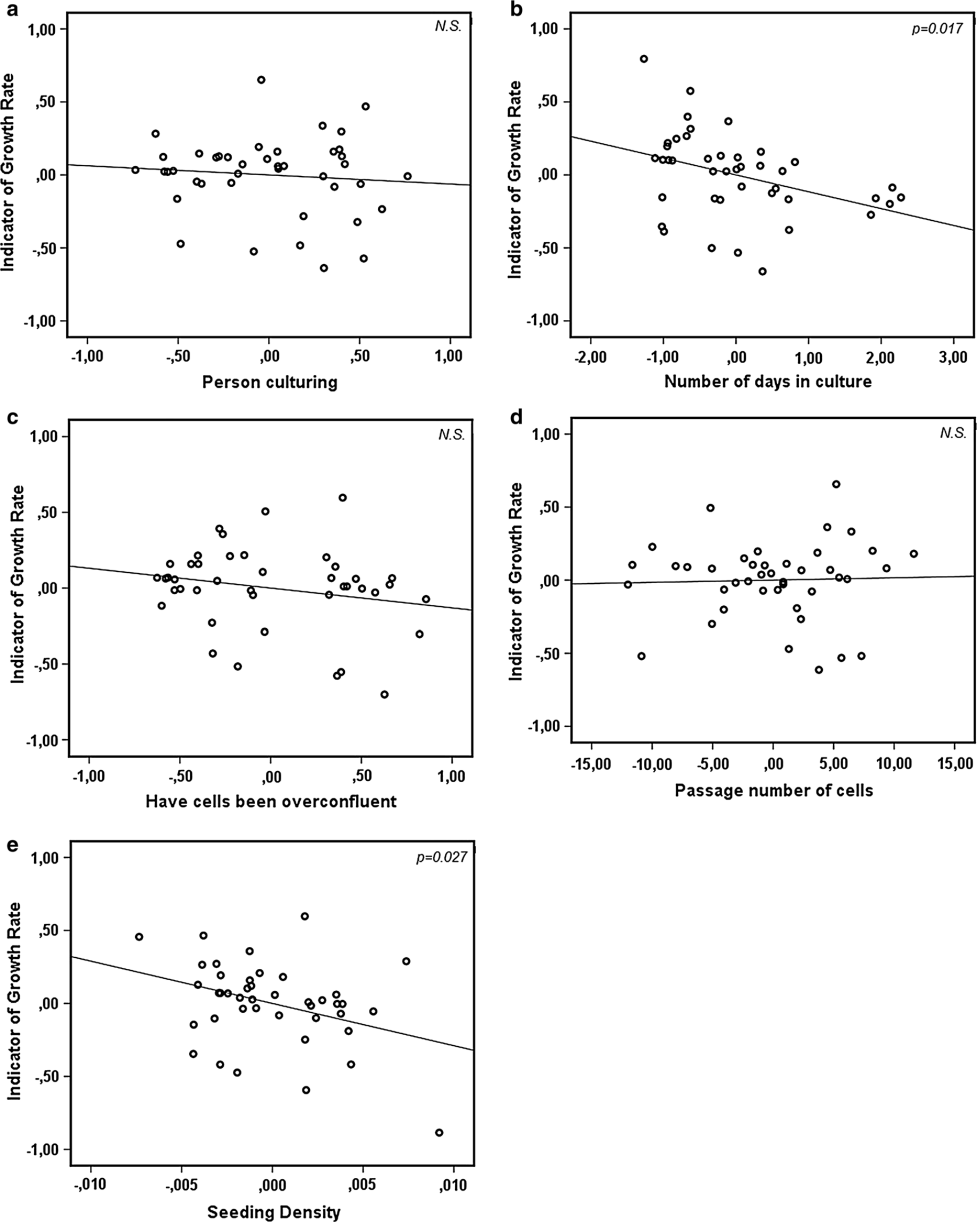
Partial regression plots of all five predictors in the exploratory linear regression model. In these plots the gradient of the regression line is equivalent to the standardized β coefficient of the predictor in the model, and signifies the correlation between cell growth and each predictor, when all other predictors are held constant. The axes display the residuals of the indicator of growth rate and the predictors. The significant negative correlations between indicator of growth rate and both number of days in culture (**b**) and seeding density (**e**) is visible, while no clear correlation is visible between indicator of growth rate and person culturing (**a**), previous overconfluency (**c**) and passage number (**d**)

The overall model accounted for 21.1 % of the variability in the indicator of growth rate in the sample tested, as indicated by the R^2^ value 0.211 in Table 1. The adjusted R^2^ value was 0.102, therefore the model only accounts for 10.2 % of variability of the indicator of growth rate of the entire population of IM-FEN cells. The model only marginally improves the prediction of the indicator of growth rate compared to the mean, indicated by the F ratio 1.931. This improvement was not significant (*p* = 0.113). However, there were two predictors that had a significant negative relationship with the indicator of growth rate, being ‘Number of days in culture’ and ‘Seeding density’ (Table 1; Fig. 2b, e). ‘Number of days in culture’ had a significant negative standardized β coefficient of −0.407 (*p* = 0.017) and a confidence interval not containing zero, meaning the negative relationship between the indicator of growth rate and ‘number of days in culture’ is statistically significant when all other variables are held constant (Table 1). ‘Seeding density’ had a significant negative standardized β coefficient of −0.393 (*p* = 0.027), and a confidence interval not containing zero, meaning the negative relationship between the indicator of growth rate and ‘seeding density’ is statistically significant when all other variables are held constant (Table 1). The correlations between the indication of growth rate and each individual predictor is visualized in Fig. 2a–e.

**Table 1:** Results of the exploratory linear regression model

Variable	Unstandardized B coefficient	Standardized β coefficient	Significance	Lower bound 95 % CI	Upper bound 95 % CI
Passage number	0.002	0.032	0.841	−0.014	0.017
Previous overconfluency	−0.131	−0.217	0.188	−0.329	0.067
Number of days in culture	−0.116	−0.407	0.017*	−0.209	−0.022
Person	−0.063	−0.103	0.572	−0.285	0.160
Seeding density	−28.951	−0.393	0.027*	−54.486	−3.417

The relationship between the indicator of growth rate of proliferating IM-FEN cells and five predictors was analyzed. ‘Number of days in culture’ and ‘Seeding density’ showed a significant effect, while the other predictors and the model overall did not

R^2^ = 0.211, adjusted R^2^ = 0.102, *F ratio* = 1.931, sig. *F ratio* = 0.113

* *p* < 0.05

**Fig. 3:**
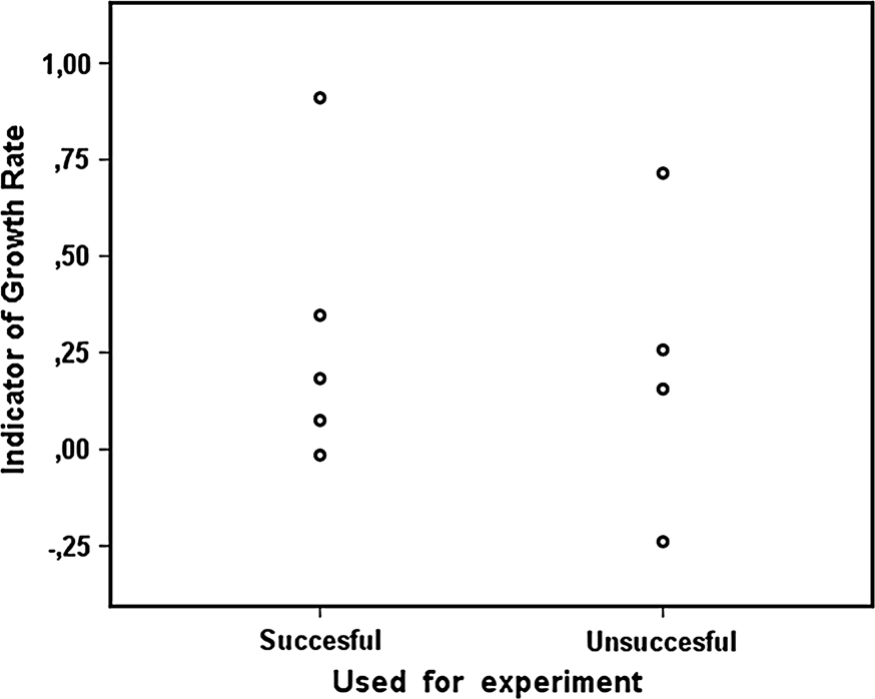
The indicator of growth rate of IM-FEN cells during proliferation does not affect the final fate of the differentiated cells. The spread of cell growth rates in both groups is very similar, and the difference between groups is not significant (*p* = 0.905)

There was no difference in the indicator of growth rate during proliferation between differentiated cells that were either successfully used in an experiment, or cells that died prematurely during differentiation (*p* = 0.905) (Fig. 3).

## Discussion

Upon introduction of the ENS cell line in our laboratory, differentiated IM-FEN cells were tested for the expression of the four neuronal proteins HuD, peripherin, tubulin, and PGP9.5 (Anitha et al. 2008); the expression of all four proteins was confirmed. Two proteins, HuD and peripherin, were expressed strongly in the samples. Tubulin was also expressed, but at low levels. PGP9.5 staining was also present in the samples, unfortunately it could not be determined if this staining was strong because the image was blurry. These results provided evidence that differentiated IM-FEN cells express a neuronal phenotype, and gave the confidence that future experiments with these cells would be performed on properly differentiated neuron-like cells.

We did not stain undifferentiated cells for neuronal markers. However, in the original publication (Anitha et al. 2008) it was shown that the expression of neuronal proteins changes gradually over time between the proliferating (33 °C) and differentiating (39 °C) conditions. Some of the parent IM-FEN cells do express neuronal markers in proliferating conditions. It has not yet been determined whether these individual cells are still proliferating. In future studies this could be determined using tracer studies. At this moment it can be stated that in a cell population of IM-FEN cells proliferation decreases as differentiation increases.

Enrichment of proliferating cells could potentially be achieved through immunoselection for proliferation markers, but this has not yet been done. There has been no need for the enrichment of IM-FEN cells, since the proliferation is high at 33 °C, when the cells are cultured in N2-medium in the presence of IFN-γ.

We have struggled with unstable growth rates of proliferating IM-FEN cells, and in this paper we have tried to find a solution. Being able to predict the growth rate of the cells would help to plan future experiments more efficiently, to build up a bank of frozen cells, to judge the health of the cells by their growth rate, and to prevent overconfluency. The analysis of five potential predictors yielded a non-significant model for the prediction of an indicator of growth rate. However, two of the chosen predictors were useful for the prediction of the indicator of growth rate; days in culture and seeding density were significant negative predictors of indicator of growth rate, meaning that more days in culture or a higher seeding density were significantly related to a lower indicator of growth rate.

Number of days in culture showed the strongest negative correlation with the indicator of growth rate. The most likely explanation for the negative correlation is that slow growing cells are culture in the same flask for a longer time, because it takes the cells longer to reach a high level of confluency. However, the causality of this relationship was not clear. Therefore, it is not clear whether culturing in the same flask for more than the average number of days (3–4 days) should be avoided. This can be tested experimentally by passaging cells with different growth rates at different schedules, before any final conclusion can be drawn concerning causality.

Seeding density also showed a significant negative correlation with the indicator of growth rate. The most likely explanation is that slow growing cells get passaged at a higher density, to support their growth. Another explanation could be that a drop in reactive oxygen species reduces growth rate at higher densities (Limoli et al. 2004). However, the causality of this relationship is unclear as well. The effect of seeding density on growth rate should again be determined empirically, including measurements of the level of reactive oxygen species, to differentiate between both explanations. However, if a lower growth rate of the previous culture indeed entices the researcher to seed at a higher density and to culture for a longer time, from these data it seems clear that these approaches do not resolve the problem of unstable cell growth. Therefore culturing slow growing cells at higher density than average (the average being about 1 million cells per 75 cm^2^ flask, or 0.0133 million cells per cm^2^) is not recommended, since it does not seem to improve growth rate. Leaving slow growing cells for many days in the same culture flask (until they become 80 % confluent, and will be passaged) does not seem to speed up the growth rate either, but is nonetheless recommended, because passaging the cells at an earlier moment will only increase their passage number and will not increase their growth rate, therefore patience is the best course.

Increasing passage number did not affect the indicator of growth rate of IM-FEN cells, making it possible to keep them in culture for a long time. Regardless, it is generally advisable to work with cells as young as possible, since over time cells potentially accumulate deviations from the original source (Hughes et al. 2007; Balls et al. 2006) and this may influence experimental results. Passage number is an indicator for the ‘age’ of the cells, their distance from the original source. A more accurate estimate is the ‘population doubling number’, however we did not have these data available. Therefore ‘passage number’ has been used in our model.

Previous overconfluency in the same continuous culture did not affect the indicator of growth rate, although the growth rate in the first culture after overconfluency is usually low (personal observation). It appears that the cells were able to recover from overconfluency, which allowed subcultures of cells from flasks that grew faster than expected and became overconfluent as a result. Nonetheless, it is good cell culture practice to passage the cells when they have not yet reached overconfluency, but are rather in the logarithmic phase of growth (Phelan 2007), so the growth of the cells is not inhibited by cell-cell contact. Additionally, overconfluent cells underwent an unintended and uncontrolled selection process where some cells died off during the overconfluency, while more sturdy cells were still alive and able to proliferate in a new flask. Therefore it is advisable to prevent overconfluency.

Finally, the person culturing did not predict the indicator of growth rate, showing that experience or subtle differences in culturing techniques did not affect growth rate.

Although two variables relevant to growth rate were found, three variables were irrelevant. To improve the model, the three irrelevant variables can be removed, and other potential variables involved in growth rate can be added. Variables linked to the culture conditions of the cells (for example the culture medium) are interesting to include in the model (Phelan 2007; Balls et al. 2006). Testing these variables was not possible in our model, because we did not have data on these factors detailed in the laboratory journals. The general practice for these factors is known, and can be used to make predictions for their potential as predictors for growth rate. The culture medium was always prepared according to the protocol, using fresh ingredients; therefore the expected variability in the medium is limited. Nonetheless, in future experiments the batch and freshness/age of the medium and all its ingredients should be listed since no batch is exactly the same and medium deteriorates over time (Phelan 2007; Balls et al. 2006). When these data are collected they can be tested as predictors for cell growth. The culture flasks in our laboratory are bought from Greiner Bio-One and are used for the culture of different cell lines and primary cells, without problems. This is an unlikely source of variation in cell culture and is therefore not interesting as a potential predictor in future experiments.

It should be noted that this was a preliminary study, and the sample size was only 42. Therefore the current model should be interpreted with caution, and results should be confirmed in other, larger samples. Although the sample size was small, our preliminary study does offer insight into the potential factors influencing the growth rate of IM-FEN cells. Researchers using these cells in future experiments can build on these results with new analyses, to be determine which (if any) additional factors can predict growth rate in proliferating IM-FEN cells. It could be an inherent feature of these cells to have changeable growth rates. The unstable growth rate has also been noted in the laboratory where the cells were originally developed (personal communication, Prof. Dr. Shanthi Srinivasan); therefore it is possible that these cells are unstable by nature, resulting in an unknown amount of variability of growth rate. It is noteworthy that in this other laboratory two adaptations have been made to the culture conditions: the CO_2_ concentration at 33 °C during proliferation has been reduced from 10 to 5 %, and the flasks and plates containing cells are placed close to the walls of the incubators to keep the temperature as stable as possible. Since these adaptations were introduced in the laboratory, the growth rate of the cells has been more stable. Although causality cannot be proven, it could be that these changes are beneficial to IM-FEN cells. Therefore, these changes are recommended for other laboratories culturing these cells. On the other hand, in Chinese hamster ovarian cells there was no significant difference between the growth rates of mother and daughter cells in 13 out of 23 monoclonal cultures, indicating growth rate can be inherited from mother to daughter cells (Davies et al. 2013), giving support to the idea that unstable growth rates might be an inherited trait in the IM-FEN cell line.

For the 42 cases used to analyze growth rate we also analyzed the fate of the cells. Twenty-one percent was used for differentiation, as most cells were used for continuous proliferation or freezing for storage and future use. Of the 42 cases 11.9 % were eventually used for experiments, while 9.5 % died in the differentiation process of unknown causes. No relationship was found between the indicator of growth rate during proliferation and cell fate during differentiation. This means that cells should not be judged on their usefulness for differentiation based on their growth rate during proliferation. However, when interpreting these results it should be kept in mind that no causal relationship was tested in this analysis, and only 9 cases were included. More data should be collected on this topic to gain more confidence in the conclusion that there is no relationship between growth rate and cell fate during differentiation.

## Conclusion

The culture of IM-FEN cells has been successfully implemented in our laboratory, resulting in properly differentiated neuron-like cells. These cells are considered valuable and suitable for further experiments based on the results presented here.

The culture of IM-FEN cells has not been without problems. Especially the proliferation stage of the IM-FEN cells has been challenging, with unexpected increases and decreases in growth rate. Two factors significantly predicting the indicator of growth rate in our statistical model were days of culture and seeding density. The causality between these predictors and growth rate remains to be determined, however a recommendation is already made not to seed slow growing cells at an increased or decreased density, and to wait until 80 % confluency before passaging cells, even if this take many days for slow growing cells. Three factors were unrelated to indicator of growth rate (person culturing, passage number, previous overconfluency) and can be excluded from future models, while factors related to the culture medium could be added in an effort to predict growth rate of IM-FEN cells in the future.

## Abbreviations

ENS: Enteric nervous system
IM-FEN: Murine immorto fetal enteric neuronal cell line
IFN-γ: Interferon-γ
PGP9.5: Protein gene product 9.5
DMEM/F12: Dulbecco’s modified Eagle’s medium: nutrient mixture F12
GDNF: Glial cell line derived neurotrophic factor
FCS: Fetal calf serum
